# Total magnetic resonance imaging of cerebral small vessel disease burden predicts dysphagia in patients with a single recent small subcortical infarct

**DOI:** 10.1186/s12883-021-02518-9

**Published:** 2022-01-03

**Authors:** Lulu Zhang, Xiang Tang, Yidan Li, Juehua Zhu, Dongxue Ding, Yun Zhou, Shanshan Diao, Yan Kong, Xiuying Cai, Ye Yao, Qi Fang

**Affiliations:** 1 Department of Neurology, First Affiliated Hospital of Soochow University, No. 899, Pinghai Road, Suzhou, 215006 Jiangsu China; 2 Department of Biostatistics, School of Public Health, Fudan University, No. 130 Dongan Road, Shanghai, 200032 China; 3 National Clinical Research Center for Aging and Medicine, Huashan, Shanghai, China; 4 Key Laboratory of Public Health Safety of Ministry of Education, Fudan University, Shanghai, China; 5 Hospital, Fudan University, No 12 Wulumuqi Zhong Road, Shanghai, 200040 China

**Keywords:** Acute ischemic stroke, Recent small subcortical infarct, Magnetic resonance imaging, Small vessel disease, Post-stroke dysphagia

## Abstract

**Background:**

This study was performed to identify the association between the total magnetic resonance imaging burden of small vessel disease and the occurrence of post-stroke dysphagia in patients with a single recent small subcortical infarct (RSSI).

**Methods:**

We retrospectively identified all patients with a magnetic resonance imaging-confirmed single RSSI. The water-swallowing test and volume-viscosity swallow test were performed within the first 24 h following admission to assess swallowing. Demographic and clinical data were extracted from our stroke database. Based on brain magnetic resonance imaging, we independently rated the presence of cerebral microbleeds, lacunes, white matter hyperintensities and enlarged perivascular spaces. The presence of each small vessel disease feature was summed to determine the total small vessel disease burden, ranging from 0 to 4.

**Results:**

In total, 308 patients with a single RSSI were enrolled. Overall, 54 (17.5%) were diagnosed with post-stroke dysphagia. The risk factors related to post-stroke dysphagia included the following: older age, higher National Institute of Health Stroke Scale scores, higher C-reactive protein level and higher fibrinogen level. Based on multiple logistic regression, National Institute of Health Stroke Scale scores and total small vessel disease burden were independent risk factors of post-stroke dysphagia in patients with a single RSSI, after adjusting for age, gender, history of hypertension, C-reactive protein level and fibrinogen level.

**Conclusions:**

Dysphagia in patients with a single RSSI was associated with a more severe total small vessel disease burden as reflected by MRI. Total MRI of cerebral small vessel disease burden may predict dysphagia in these patients. Furthermore, more severe total small vessel disease burden was associated with systemic inflammation.

## Introduction

Post stroke dysphagia (PSD) is defined as difficulty in swallowing after stroke. PSD is a common disabling symptom associated with pneumonia, malnutrition and poor clinical outcomes [1]. PSD rates are particularly high among acute ischemic stroke (AIS) patients who are older, or have severe stroke and larger infarcts [2]. Brainstem strokes represent another risk factor for dysphagia and result in the most severe impairment of swallowing [3].

However, PSD also occurs in up to one-fifth of patients with a single recent small subcortical infarct (RSSI) [4]. These rates indicated the necessity for standardized swallowing assessment in patients with RSSI, at least through bedside screening, to avoid potentially severe complications. RSSI was previously defined as lacunar infarct and was noted in 25% of all AIS patients [5]. RSSI is thought to result from the occlusion of a small, single perforating artery supplying subcortical areas, such as the basal ganglia, thalamus, centrum semiovale and pons [6]. The exact mechanisms explaining the occurrence of PSD following RSSI are not well documented and have not been validated in larger cohorts. A prior retrospective study reported that PSD in supratentorial RSSI may result from bilateral pyramidal tract damage caused by pre-existing contralateral lesions (including lacunes or confluent white matter hyperintensities (WMHs)) [1]. Another study identified severe stroke, pontine infarcts and severe WMHs as risk factors for swallowing dysfunction in patients with a single RSSI [4].. Detailed data on the occurrence of swallowing dysfunction in RSSI patients are lacking. No studies have specifically investigated PSD in RSSI patients in the context of total SVD burden.

Multiple subcortical regions were also associated with abnormal swallowing, which may provide an interconnection between cortical swallowing centers and the central pattern generator [7]. These anatomical structures and pathways may be disrupted not only by RSSI but also by coexisting damage from small vessel disease (SVD) [8]. The term “cerebral SVD” describes a range of neuroimaging features, that have long been implicated in cognitive impairment, stroke and gait disorder [5]. Magnetic resonance imaging (MRI) markers of SVD include cerebral microbleeds (CMBs), lacunes, WMHs, enlarged perivascular spaces (ePVS) and brain atrophy [9]. The exact mechanism of SVD is not well known, but this condition is thought to result from damage to perforating cerebral arterioles, capillaries, and venules, which ultimately cause brain damage, including cerebral white matter and deep gray matter [5]. Importantly, inflammation has been increasingly implicated as a prominent component and a candidate factor of SVD [10]. Markers of inflammation are classified as systemic inflammation (e.g., C-reactive protein, interleukin-6 and fibrinogen) and vascular inflammation/endothelial dysfunction (e.g., homocysteine, von Willebrand factor, and Lp-PLA2, [11]. Existing studies have indicated that systemic and vascular inflammation/endothelial dysfunction are differentially associated with different forms of SVD. Specifically, vascular inflammation was related to hypertensive arteriopathy-type SVD, whereas systemic inflammation was related to cerebral amyloid angiopathy (CAA)-type SVD. The most widely investigated markers of inflammation include C-reactive protein (CRP) and homocysteine. CRP is a sensitive but nonspecific marker of systemic inflammation. Homocysteine is thought to cause damage to the endothelium [12] and subsequently result in blood–brain barrier dysfunction [13]. Associations between vascular clinical factors and SVD remain controversial. Hypertension, smoking, diabetes and sex were all previously proposed to be associated with SVD [14]. However, conclusions were inconsistent not only among different studies [15] but also among different individual features of SVD [16, 17].

The total SVD burden combines 4 individual but closely correlated MRI features of SVD in one measure, including CMBs, lacunes, WMHs and ePVS [5, 18], providing a complete estimate of the full impact of SVD in a simple manner. The total SVD burden reflects the overall impact of SVD on neurological diseases in a more complete and pragmatic manner than only 1 or 2 individual features could [19, 20].

## Methods

### Participants

We retrospectively identified all AIS patients in our stroke database who were admitted to the stroke unit at First Affiliated Hospital of Soochow University between October 2017 and January 2019. The selection criteria were as follows: (1) diagnosis of AIS confirmed by diffusion-weighted MRI (DWI); and (2) diagnosis of a single RSSI according to the STandards for ReportIng Vascular changes on nEuroimaging (STRIVE) consensus criteria [21] by two neuroimaging experts blinded to the clinical data. In case of disagreement, a meeting was held to establish consensus. If the two experts still disagreed, we invited a third expert and adopted the majority opinion. Patients with the following were excluded: (1) multiple RSSIs or additional acute infarcts in other locations; (2) pre-existing dysphagia or concomitant diseases likely to cause dysphagia, including dementia; (3) concomitant brain hemorrhage; (4) brain tumors; and (5) severe hepatic or renal dysfunction or end-stage severe disease. All patients were divided into two groups according to the following swallowing assessment: (1) patients with PSD; and (2) patients with no PSD.

Each participant in our stroke database was asked to sign two copies of an informed consent document; one copy was kept in the stroke center office and was also scanned and saved in PDF format. Consent was obtained separately for four components: nonblood biomarkers, blood samples, image acquisition and storage of blood samples for future analyses. The ethics committee of our hospital approved the study protocol. Informed consent was obtained from all patients or their relatives upon admission.

### Swallowing assessment

Swallowing function was examined within the first 24 h following admission before oral feeding using the water-swallowing test (WST) and volume-viscosity swallow test (V-VST); both tests were performed by a trained neurologist blinded to the clinical data. The WST was performed using 30 ml of water while sitting at a 90°angle [22]. The V-VST was assessed with gradually increasing volumes of 5, 10 to 20 ml and with different viscosities (thin liquid, nectar-like and spoon thick) in combination with a pulse-oximeter to evaluate both the efficacy and the safety of swallowing function. Signs of impaired efficacy of swallowing included an inefficient labial seal, oral residue, fractional swallowing and pharyngeal residue. Signs of impaired safety of swallowing include changes in voice quality, coughing and a decrease in oxygen saturation (SpO_2_) ≥3% for > 1 min compared to baseline [23]. Patients who presented any sign of impaired efficacy and/or safety when swallowing were considered positive for PSD.

### Clinical data

We extracted all the following variables: age, sex, systolic blood pressure, diastolic blood pressure, medical history (including hypertension, diabetes mellitus, atrial fibrillation, smoking and previous stroke), clinical data on admission (including relevant laboratory indicators, stroke severity measured by NIHSS, and thrombolytic treatment) and markers of inflammation (including C-reactive protein, fibrinogen, homocysteine and Lp-PlA2 levels). The diagnosis of post-stroke pneumonia was identified by our treating team and defined based on ≥3 of the following 6 features: (1) fever (> 38 °C); (2) productive cough; (3) abnormal respiratory examination; (4) abnormal chest radiograph; (5) white blood cell count> 12,000/ml; and (6) isolation of a relevant pathogen and use of antibiotics.

### MRI acquisition

All patients were scanned in a 3 T MR scanner (MAGNETOM Skyra; Siemens Healthineers, Erlangen, Germany). A 20-channel brain array coil was used for signal reception. The images obtained included transverse T1-weighted turbo spin-echo (TSE) images (repetition time (msec)/echo time (msec), 700/14; section thickness, 3 mm; intersection gap, 0.5 mm; field of view, 25 cm; matrix, 384 × 336), transverse T2-weighted TSE images (repetition time (msec)/effective echo time (msec) 6000/124; section thickness, 3 mm; intersection gap, 0.3 mm; field of view, 25 cm; matrix, 384 × 336), fluid-attenuated inversion recovery (FLAIR) turbo spin-echo (TSE) images (repetition time (msec)/echo time (msec), 7000/78; inversion time (msec) 2215; section thickness, 5 mm; intersection gap, 1 mm; field of view, 220 cm; matrix, 320 × 320), and susceptibility-weighted gradient echo (GRE) images (repetition time (msec), 27; inversion time (msec) 20; flip angle 15°; section thickness, 1.5 mm; intersection gap, 0.3 mm; field of view, 220 cm; matrix, 256 × 256). DWI was obtained to calculate an apparent diffusion coefficient using a 2D echo planar imaging sequence with multiple b-value acquisitions (0, 100, 800, 1000 and 1500 s/mm2), with the diffusion-sensitizing gradients applied along the X, Y and Z axes. All enrolled patients were admitted, and brain MRI was performed within 3 days after symptom onset (mean 2.35 ± 0.73 days and 2.48 ± 0.82 days, range 1–3 days). Brain lesions were localized according to hemisphere (left, right).

### MRI analysis

**Fig. 1:**
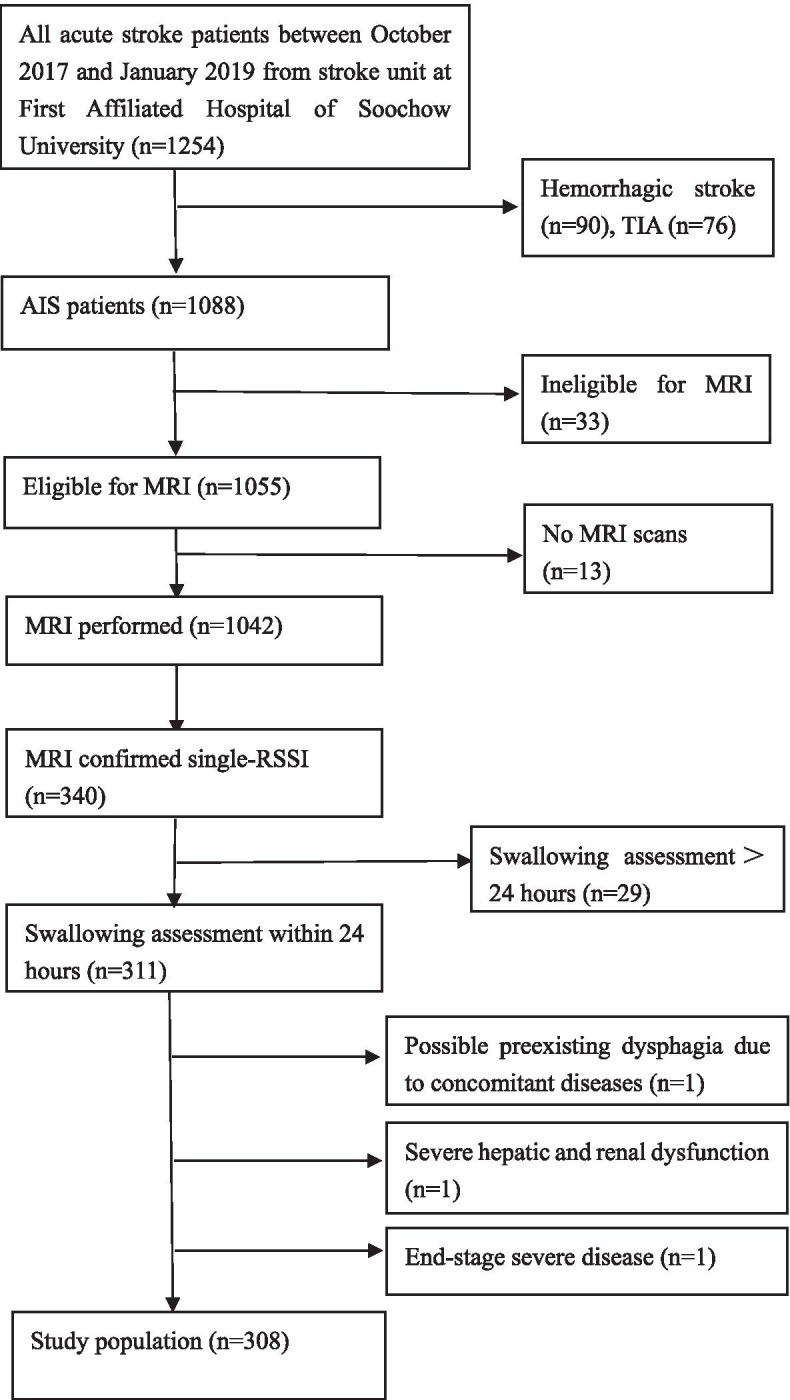
Examples for the four different single recent small subcortical infarct locations on diffusion-weighted magnetic resonance imaging: basal ganglia (**A**), thalamus (**B**), centrum semiovale (**C**) and pons (**D**)

All images were assessed by two neuroradiologists blinded to the clinical information. In case of disagreement, a meeting was held to establish a consensus. A single RSSI was identified based on STRIVE criteria [21] and located in four brain regions as follows: basal ganglia, thalamus, centrum semiovale and pons [24] (Fig. 1). Briefly, CMBs were defined based on SWI as small (< 5 mm), homogeneous, rounded lesions of low signal intensity [21, 25]. Lacunes were identified as asymptomatic rounded or ovoid hyperintense lesions in subcortical areas between 3 and 20 mm in diameter, of equal signal intensity to CSF on T2 and FLAIR, with a hyperintense rim on FLAIR and no increased signal on DWI [26]. Deep and periventricular WMH were graded according to the Fazekas score from 0 to 3 [27]. EPVS were defined as small (< 3 mm) punctate (if perpendicular to the plane of the scan) and linear (if parallel) lesions in both the BG and centrum semiovale regions with signal intensity equal to that of cerebrospinal fluid on T1, T2 and FLAIR sequences without a hyperintense rim on FLAIR images [21].

### Total MRI-confirmed SVD burden

**Fig. 2:**
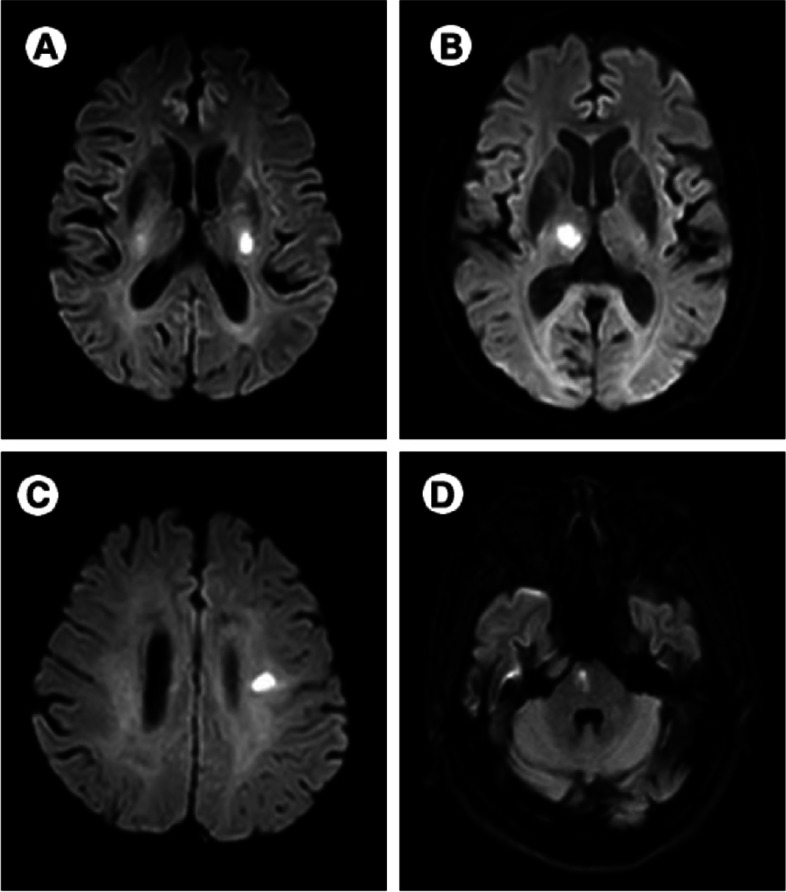
Examples of the four different features of MRI-confirmed small vessel diseases: lacune (**A**), cerebral microbleeds (**B**), enlarged perivascular spaces (**C**), and white matter hyperintensities (**D**)

We constructed the total MRI-confirmed SVD burden on an ordinal scale ranging from 0 to 4 by counting 4 MRI features of SVD (CMBs, lacunes, WMH and ePVS) [16]. The presence of each of the following items was awarded one point (Fig. 2): presence of lacunes and CMBs were defined as ≥1 asymptomatic lacune or ≥ 1 CMB (1 point if present); moderate to extensive (≥11) PVS (1 point if present); presence of WMH was defined as either deep WMH (Fazekas score 2 or 3) or periventricular WMH (Fazekas score 3) (1 point if present) [28].

### Statistical analysis

All values were analyzed using SPSS 20.0 software (SPSS, Inc., Chicago, IL, USA). Continuous data are compared by the t-test or Mann-Whitney U test and are shown as the mean ± SD, minimum and maximum values in patients with dysphagia and controls with statistical significance. Categorical data differences in patients with dysphagia and controls are represented with statistical significance based on the chi-squared test or Fisher’s exact test to describe associations between radiological factors and PSD. Binary univariate and multivariate logistic regression analyses with simultaneous inclusion were applied to identify predictors of dysphagia and compare clinical outcomes in participants with and without dysphagia. Pearson correlations were conducted to analyze the relationships between markers of inflammation and the total SVD burden.

## Results

### Patient characteristics

**Fig. 3:**
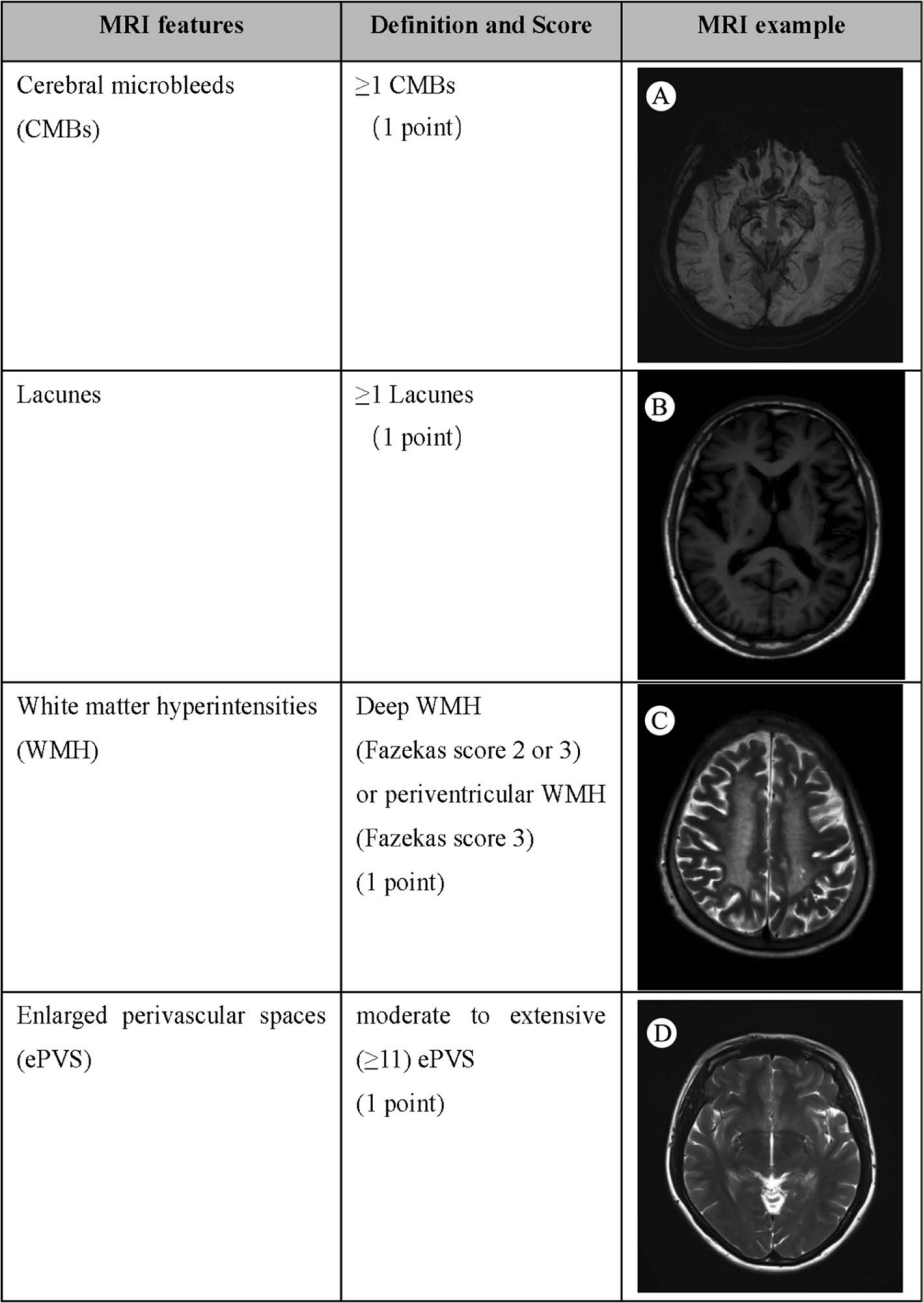
Details of study recruitment. AIS: acute ischemic stroke; TIA: transient ischemic attack

A total of 308 patients fulfilled the inclusion criteria and were included in the study (Fig. 3). 54 (17.53%) of them were with PSD. Demographic and clinical data of the study population and differences between dysphagic and non- dysphagic patients with a single RSSI are displayed in Table 1. The groups significantly differed in age: patients with dysphagia were older than non-dysphagic patients (67.54 ± 11.74 vs. 61.00 ± 13.06 years, *p* = 1.00 × 10^− 3^). Patients in the non-dysphagia group exhibited higher triglyceride (1.67 ± 1.00 vs. 1.33 ± 0.51 mmol/l, *p* < 1.00 × 10^− 3^) and uric acid (312.56 ± 91.35 vs. 282.17 ± 80.91 μmol/l, *p* = 0.03) levels than those in the dysphagia group. PSD patients exhibited higher NIHSS scores (7.78 ± 6.23 vs. 3.23 ± 2.92, *p* < 1.00 × 10^− 3^). A significant difference in post-stroke pneumonia was found between the groups, with dysphagic patients exhibiting a higher incidence (44.40% vs. 14.96%, *p* < 1.00 × 10^− 3^). A significantly strong association was noted between both higher CRP (62.96% vs. 27.95%, *p* < 1.00 × 10^− 3^) and fibrinogen levels (3.18 ± 0.96 vs. 2.61 ± 0.93, *p* < 1.00 × 10^− 3^).

**Table 1:** Demographic and clinical data of single-RSSI patients with dysphagia and controls

Demographic and Clinical Data	Dysphagia (*n* = 54)	Controls (*n* = 254)	t/Z/χ^2^	*P*
Age (years)	67.54 ± 11.74, 42.00–91.00	61.00 ± 13.06, 22.00–89.00	t = − 3.40	1.00 × 10^− 3^
Gender male/female	37/17	176/78	χ^2^ = 0.01	0.91
Systolic Blood Pressure (mmHg)	152.57 ± 24.87, 105.00–220.00	147.44 ± 21.40, 100.00–230.00	t = − 1.55	0.12
Diastolic Blood Pressure (mmHg)	82.54 ± 12.11, 53.00–111.00	81.94 ± 12.89, 50.00–131.00	t = − 0.31	0.76
History of Hypertension (yes/no)	40/14	192/62	χ^2^ = 0.06	0.81
History of Diabetes (yes/no)	17/37	75/179	χ^2^ = 0.08	0.78
Smoking yes/no	12/42	68/186	χ^2^ = 0.48	0.49
History of AF^a^ (yes/no)	4/50	7/247	χ^2^ = 2.80	0.09
Previous Stroke (yes/no)	12/42	34/220	χ^2^ = 2.74	0.10
Triglyceride (mmol/L)	1.33 ± 0.51, 0.49–2.55	1.67 ± 1.00, 0.38–9.11	t = 3.68	< 1.00 × 10^− 3^*
Total Cholesterol (mmol/L)	4.30 ± 1.03, 1.51–6.33	4.36 ± 1.05, 1.94–9.90	t = 0.38	0.70
LDLC (mmol/L)	2.62 ± 0.94, 0.63–5.01	2.65 ± 0.91, 0.65–8.11	t = 0.25	0.80
Creatinine (μmol/L)	67.78 ± 13.99, 47.30–102.00	70.24 ± 21.53, 33.20–225.30	Z = 0.34	0.73
Uric Acid (μmol/L)	282.17 ± 80.91, 138.40–472.40	312.56 ± 91.35, 92.50–648.00	t = 2.24	0.03
Fasting Blood Glucose (μmol/L)	6.03 ± 1.84, 3.92–12.76	5.98 ± 2.07, 3.34–19.60	Z = − 0.96	0.34
Homocysteine^b^ (μmol/L)	14.13 ± 9.12, 6.20–55.60	12.84 ± 8.67, 3.40–74.40	Z = − 1.33	0.18
Hemoglobin A1c^b^ (%)	7.08 ± 1.79, 5.20–12.10	6.72 ± 1.67, 4.90–15.10	Z = − 1.14	0.25
NIH Stroke Scale	7.78 ± 6.23, 0.00–36.00	3.23 ± 2.92, 0.00–15.00	Z = − 6.34	< 1.00 × 10^− 3^*
Higher CRP^a^	34/20	71/183	χ^2^ = 24.29	< 1.00 × 10^− 3^*
Fibrinogen (g/l)	3.18 ± 0.96, 0.75–6.18	2.61 ± 0.93, 0.75–6.77	t = − 4.06	< 1.00 × 10^− 3^*
Lp-PlA2^b^ (ug/l)	139.29 ± 56.82, 63.81–311.64	157.36 ± 140.79, 56.04–800.00	Z = − 0.85	0.39
Thrombolytic (yes/no)	12/42	53/201	χ^2^ = 0.05	0.82
Post-stroke pneumonia (yes/no)	24/30	38/216	χ^2^ = 24.08	< 1.00 × 10^− 3^*

*: *p* < 0.001

^a^ AF refers to atrial fibrillation, LDLC refers to low density lipoprotein cholesterol, and higher CRP refers to C-reactive protein ≥3 mg/l.

^b^ Forty four patients with dysphagia and 215 controls took part in homocysteine tests; 37 patients with dysphagia and 196 controls took part in hemoglobin A1c tests; 17 patients with dysphagia and 68 controls attended Lp-PlA2 tests

Continuous data are compared by the t-test or Mann-Whitney U test and are shown as the mean ± SD, minimum and maximum values in patients with dysphagia and controls with statistical significance. Categorical variables between patients and controls are represented with statistical significance based on the chi-squared test (χ^2^ & p) or Fisher’s exact test (Z & p)

### Associations between radiological factors and PSD

The majority of single RSSIs were located in the basal ganglias (*n* = 142, 46.10%) followed by pons (*n* = 70, 22.73%), centrum semiovales (*n* = 60, 19.48%), and thalamus (*n* = 36, 11.69%). Neuroimaging examples of prespecified single-RSSI locations are provided in Fig. 1. Given that single-RSSI locations exhibited significant differences between PSD and non-PSD patients (*p* = 0.04), we further investigated the associations of single-RSSI locations and dysphagia as shown in Table 2. Patients with RSSI located in the pons suffered from dysphagia more often (OR = 2.95, *p* = 0.50 × 10^− 3^), whereas no significant differences were noted between patients with RSSI located in the basal ganglia, thalamus and centrum semiovale (OR = 0.91, *p* = 0.75; OR = 0.00, *p* = 1.00; OR = 0.47, *p* = 0.23, respectively). No associations were noted between the laterality of the single RSSI and dysphagia (left, 57.41% vs. 53.15%; *p* = 0.57). All four MRI features of SVD (including lacunes, perivascular spaces, WMH Fazekas 2–3 and microbleeds) were significantly different between single-RSSI patients with and without dysphagia, as shown in Table 2.

**Table 2:** Radiological data of single-RSSI patients with dysphagia and controls

Variable	Dysphagia (*n* = 54)	Controls (*n* = 254)	χ^2^/z	*P*
Single-RSSI Location, n (%)				
Basal Ganglia	23(42.59)	119(46.85)	χ^2^ = 0.10	0.75
Thalamus	1(1.85)	35(13.78)	z = 0	1.00
Centrum Semiovale	8(14.82)	52(20.47)	χ^2^ = 1.47	0.23
Pons	22(40.74)	48(18.90)	χ^2^ = 12.10	0.50 × 10^− 3^
Lesion in Left Hemisphere, n (%)	31(57.41)	135(53.15)	χ^2^ = 0.32	0.57
MRI features of SVD, n (%)				
Lacunes	44(81.48)	130(51.18)	χ^2^ = 16.64	< 1.00 × 10^− 3^*
Perivascular Spaces	36(66.67)	77(30.31)	χ^2^ = 25.34	< 1.00 × 10^− 3^*
WMH Fazekas 2–3^a^	26(48.15)	65(25.59)	χ^2^ = 10.89	1.00 × 10^− 3^
Microbleeds	16(29.63)	17(6.69)	χ^2^ = 24.49	< 1.00 × 10^−3^*

*: *p* < 0.001

^a^ WMH refers to white matter hyperintensities

Categorical data differences in patients and controls are represented with statistical significance based on chi-squared test (χ^2^ & p) or Fisher’s exact test (Z & p). Patients located in pons suffered from dysphagia more often (OR = 2.95, *p* = 0.50 × 10^− 3^), and there were no significant differences between patients located in the basal ganglia, thalamus and centrum semiovale (OR = 0.91, *p* = 0.75; OR = 0.00, *p* = 1.00; OR = 0.47, *p* = 0.23, respectively). There were no associations between the laterality of the single RSSI side and dysphagia (left, 57.41% vs 53.15%; *p* = 0.57). MRI features of SVD (including lacunes, perivascular spaces, WMH Fazekas 2–3 and microbleeds) were significantly different between single-RSSI patients with and without dysphagia

### Associations between total SVD burden and PSD

Among the single-RSSI patients who scored 1, the most common feature (62.61%) was lacunes followed by ePVS (26.09%), CMBs (6.09%) and WMH (5.21%) (Table 3). Among patients who scored 2, all possible combinations were present, with combinations of WMH + lacunes (34.25%) and ePVS + lacunes (34.25%) were predominant. Among patients who scored 3, all possible combinations were present, with PVS + WMH + lacunes (67.57%) was most common. Dysphagic patients had higher ratings of total SVD burden than non-dysphagic patients (Table 3; *p* < 1.00 × 10^− 3^). In multivariate logistic regression models for patients with dysphagia, total SVD burden was identified as an independent risk factor for PSD (OR = 2.27, [1.56, 3.31], *p* = 1.75 × 10^− 5^; Table 4).

**Table 3:** Total SVD score values for single-RSSI patients with dysphagia and controls

Total SVD Score	All Patients (*n* = 308)	Dysphagia (*n* = 54)	Controls (n = 254)	χ^2^	*P*
0	73(23.70)	6(11.11)	67(26.38)	5.74	0.02
1	115(37.34)	7(12.96)	108(42.52)	16.63	< 1.00 × 10^− 3^*
2	73(23.70)	16(29.63)	57(22.44)	1.27	0.26
3	37(12.01)	17(31.48)	20(7.87)	23.48	< 1.00 × 10^−3^*
4	10(3.25)	8(14.82)	2(0.79)	27.89	< 1.00 × 10^−3^*

*: *p* < 0.001

SVD refers to small vessel disease. On brain magnetic resonance imaging, we independently rated the presence of cerebral microbleeds, lacunes, white matter hyperintensities and enlarged perivascular spaces. The presence of each SVD feature was summed in the total SVD score ranging from 0 to 4. Data presented as number (%). Categorical data differences in patients and controls are represented with statistical significance based on chi-squared test (χ^2^ & p). Mann–Whitney test of dysphagia patients vs. Controls in Single-RSSI patients, Z = − 6.29, *p* < 1.00 × 10^− 3^

**Table 4:** Multivariable logistic regression model for predicting patients with dysphagia

Variables	Odds Ratio	95% CI	t	*P* value
Age	1.02	0.99,1.05	1.16	0.25
Gender	1.59	0.70,3.63	1.11	0.26
History of Hypertension	0.41	0.18,0.95	−2.08	0.04
NIH Stroke Scale score	1.25	1.13,1.37	4.46	8.12 × 10^− 6^**
Higher CRP^†^	1.66	0.77,3.59	1.29	0.20
Fibrinogen (g/l)	1.19	0.83,1.71	0.95	0.34
SVD burden^a^	2.27	1.56,3.31	4.30	1.75 × 10^−5^**

^a^ Higher CRP refers to C-reactive protein ≥3 mg/l; SVD refers to small vessel disease

### Correlations between markers of inflammation and total SVD burden

**Fig. 4:**
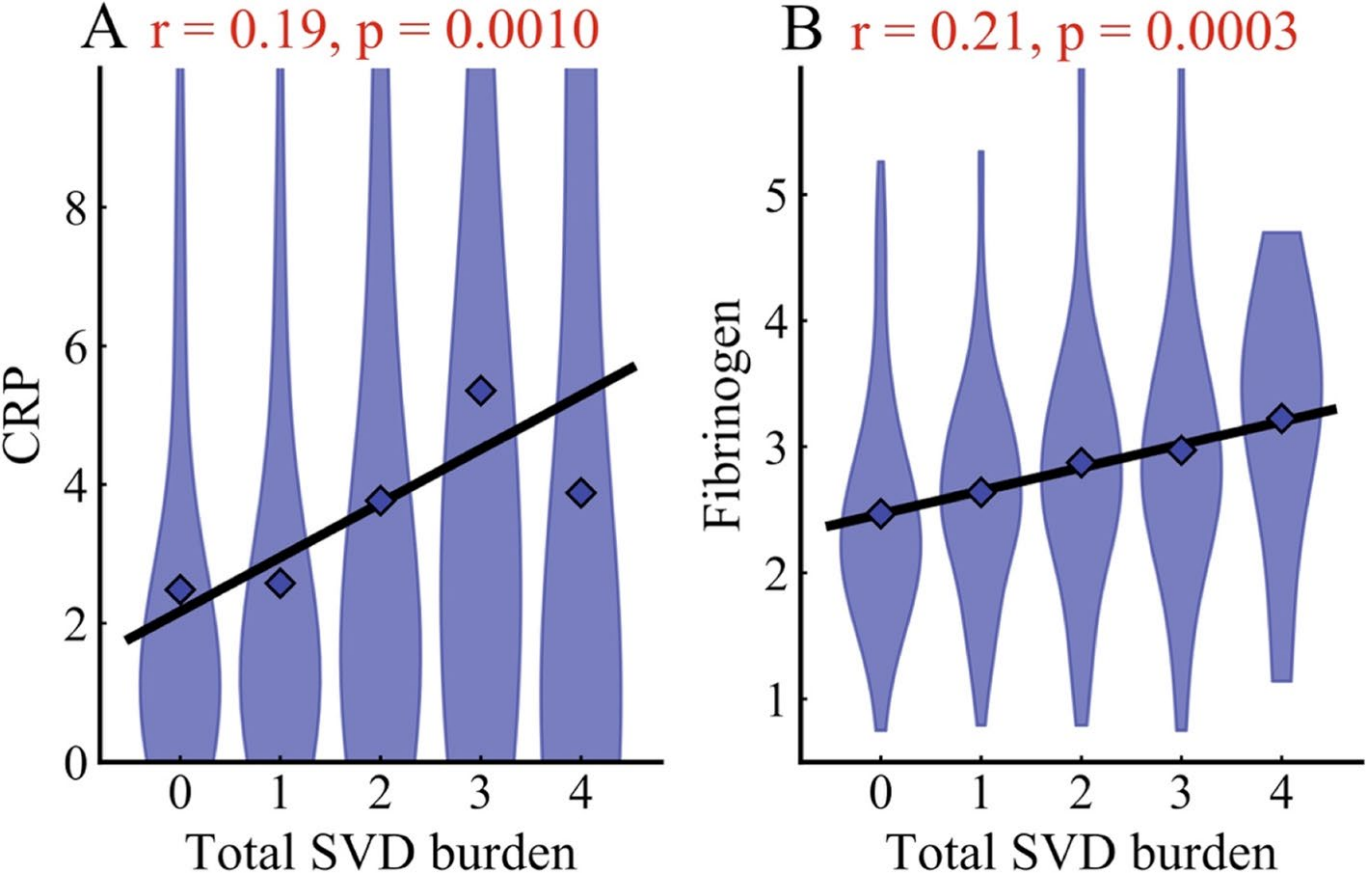
Linear correlation of plasma C-reactive protein and fibrinogen with total SVD burden. Panel A: Pearson correlations were conducted to analyze the relationships between plasma CRP and total SVD burden in single-RSSI patients (r = 0.19, *p* = 1.00 × 10^− 3^). Panel B: Pearson correlations were conducted to analyze the relationships between plasma fibrinogen and total SVD burden in single-RSSI patients (r = 0.20, *p* = 0.30 × 10^− 3^)

Table 5 presents the correlations between markers of inflammation and total SVD burden in single-RSSI patients. The CRP and fibrinogen levels were positively correlated with the total SVD burden score (r = 0.19, *p* = 1.00 × 10^− 3^; r = 0.21, *p* = 3.00 × 10^− 4^, respectively, Fig. 4). No associations between homocysteine or Lp-PlA2 levels and total SVD burden score were observed (r = 0.08, *p* = 0.22; r = 0.11, *p* = 0.30, respectively).

**Table 5:** Correlations between markers of inflammation and total SVD burden in single-RSSI patients

Variables	SVD burden score	
	r	***P*** value
CRP^a^	0.19	1.00 × 10^−3^
Fibrinogen (g/l)	0.21	3.00 × 10^−4^
Homocysteine (μmol/l) ^b^	0.08	0.22
Lp-PlA2 (ug/l) ^b^	0.11	0.30

^a^ CRP refers to C-reactive protein

^b^ Forty four patients with dysphagia and 215 controls took part in homocysteine tests, while 17 patients with dysphagia and 68 controls attended Lp-PlA2 tests

The CRP and fibrinogen levels were positively correlated with the total SVD burden score (r = 0.19, *p* = 1.00 × 10^−3^; r = 0.20, *p* = 3.00 × 10^− 4^, respectively). No associations between homocysteine or Lp-PlA2 level and total SVD burden score were observed (r = 0.08, *p* = 0.22; r = 0.11, *p* = 0.30, respectively)

## Discussion

This study showed two major findings in AIS patients with a single RSSI: (1) Clinical risk factors for PSD with a single RSSI were identified: older age, higher stroke severity (NIHSS), and elevated CRP and fibrinogen levels. (2) Dysphagia in single-RSSI patients was associated with a more severe total small vessel disease burden as reflected by MRI. Total MRI of cerebral small vessel disease burden may predict dysphagia in these patients. Furthermore, more severe total small vessel disease burden was associated with systemic inflammation.

PSD is common in hospitalized patients and associated with increased mortality and comorbidities, including post-stroke pneumonia, malnutrition, dehydration and mortality [29]. Among AIS patients, 50 to 80% had trouble swallowing, especially during the first week after their stroke [30]. The importance of screening for PSD has been emphasized at both international symposiums and in clinical audit reports [31]. The prevalence of PSD (17.53%) among AIS patients with RSSI in our study was consistent with other investigators given that 20% suffered from PSD. We also confirmed previous findings demonstrating that patients with PSD carried a high risk of post-stroke pneumonia (44.44%).

Prior studies using different methods demonstrated that bilateral activation of the sensorimotor cortex [32] and bilateral redistribution of swallowing networks after stroke [33]. In fact, many structures associated with swallowing are located in subcortical regions, such as corticonuclear tracts, periventricular connections of cortical regions and extrapyramidal pathways [8]. Damage to subcortical lesions on one hemisphere might be completely compensated by the contralateral side. However, studies identified that almost one-quarter of patients with RSSI had dysphagia. We hypothesized that PSD may be driven not only by a single RSSI but also by concomitant cerebrovascular lesions. Widely distributed morphological changes due to SVD may have a considerable effect [34]. Studies investigating RSSI patients in the context of SVD are largely unknown [35].

SVD is a common condition that affects small cerebral arterioles, capillaries, and venules. This condition has long been implicated with clinical manifestations ranging from clinically silent to focal neurological dysfunction, such as stroke, and even to global neurological symptoms and dementia [36]. Features of SVD on MRI included RSSI, WMH, lacunes, ePVS, CMBs and atrophy [37]. The terminology for these lesions has varied greatly between studies [38, 39]. Neuroimaging consensus standards for the classification of SVD were first proposed by the US National Institute of Neurological Disorders and Stroke and the Canadian Stroke Network [40]. The STandards for ReportIng Vascular changes on nEuroimaging (STRIVE) consensus defined clear, rigorous, evidence-based, and easy-to-apply terminology for SVD, which provided a consistent approach to neuroimaging [41].

In our study, we identified that NIH stroke scale and total SVD burden were independent risk factors for PSD in RSSI patients, after adjusting for age, gender, history of hypertension, C-reactive protein level and fibrinogen level. A higher NIH stroke scale was undoubtedly one of the most important predictors of dysphagia in all AIS patients. We first proposed the possible impact of the total SVD burden on swallowing in RSSI patients, which comprehensively assessed pre-existing damage to CMBs, lacunes, WMHs, and ePVS. Our study showed evidence for the great importance of the total SVD burden on PSD in RSSI patients. Previous research [30, 42, 43] has proposed various risk factors for PSD. Age, stroke severity and larger infarctions were consistently considered to be independent predictors for PSD [31, 44]. In addition to the brain stem, both cortical and subcortical regions play an important role in swallowing [3]. To date, there has been no clear conclusion about the relationship between brain lesion locations and the occurrence of PSD. However, almost none of these studies particularly focused on dysphagia in patients with RSSI.

Dysphagia in subcortical stroke may be caused by damage to swallowing pathways, including corticonuclear tracts, extrapyramidal pathways and periventricular connections of cortical regions. Prior studies investigating larger subcortical strokes reported an impact of acute lesion locations on the occurrence and severity of PSD but rarely explored the combined effects of acute and pre-existing cerebrovascular lesions on dysphagia [45]. A retrospective study revealed that PSD was closely linked to bilateral pyramidal tract damage by both acute RSSI and pre-existing contralateral cerebrovascular lesions (lacunes and severe WMH) [1]. Therefore, widely distributed morphological changes caused by SVD may additionally contribute to PSD especially for RSSI patients.

Little information is known about the pathogenesis of SVD and how this process results in neurological disease. However, the process has been attributed to proximal perforating arteriolar atheroma, lipohyalinosis, or fibrinoid necrosis [46], which were thought largely to result as a consequence of hypertension or vasospasm or recently to result from inflammation. Proximal perforating arteriolar atheroma was associated with a larger infarct of the basal ganglia and was more likely to be progressive stroke [47]. Lipohyalinosis was thought to be accompanied by additional features of SVD, such as WMHs and lacunes [48]. Only a few acute lacunar infarcts, especially basal ganglia lesions, were caused by emboli.

At present, there are no consistent conclusions about the relationship between systemic inflammation and SVD. Our data showed that the CRP and fibrinogen levels were positively correlated with the total SVD burden score. No associations between homocysteine or Lp-PlA2 level and total SVD burden score were observed. In other words, severe total SVD burden was associated with higher systemic inflammation. Longitudinal investigations demonstrated that systemic inflammation, especially if inflammation was sustained in the long term, promoted and predicted SVD progression [11]. The Atherosclerosis Risk in Communities study identified that a sustainable elevated level of CRP during midlife highly increased the risk of SVD after 20 years [49]. The existing literature revealed strong associations between SVD and markers of vascular inflammation rather than systemic inflammation in AIS patients, suggesting that vascular inflammation/endothelial dysfunction and alterations to the blood–brain barrier may be the driving force behind SVD [50]. A small number of patients with Lp-PlA2 data may account for the failure to demonstrate the association with dysphagia. Therefore, combined with previous results, this study might also confirm the possible effectiveness of anti-inflammatory treatment for post-stroke dysphagia in the future.

An important difference between our study and those of others was that we focused on RSSI patients and explored the relationship between pre-existing SVD and PSD. In addition, the total MRI burden score of SVD we used provided a more complete overall view of the pre-existing SVD than the individual features separately. Several limitations need to be further addressed. First, both WST and V-VST were assessed using bedside screening tests. Although the V-VST has been shown to be a well-validated clinical instrument with high sensitivity and specificity, instrumental testing, such as videofluoroscopic or flexible endoscopic evaluation of swallowing, might have aided in detection with higher precision and yielded higher rates of PSD. Second, further analysis of the various factors influencing the associations between inflammation and SVD (e.g., sex, ethnicity, APOE genotype, duration of inflammation) should be further assessed.

## Conclusions

In conclusion, this study showed that dysphagia occurred in approximately 20% of AIS patients with a single RSSI. Several possible clinical risk factors for PSD were identified: older age, higher stroke severity (NIHSS), and elevated levels of CRP and fibrinogen. Dysphagia in patients with a single RSSI was associated with a more severe total small vessel disease burden as reflected by MRI. Total MRI of cerebral SVD burden may predict dysphagia in these patients. Furthermore, more severe total SVD was associated with systemic inflammation.

## Data Availability

The data that supported the findings of this study were available from the corresponding author upon reasonable request.

## Abbreviations

PSD: Post-stroke dysphagia
AIS: Acute ischemic stroke
NIHSS: National Institutes of Health Stroke Scale
WST: Water-swallowing test
V-VST: Volume-viscosity swallow test
MRI: Magnetic resonance imaging
SVD: Small vessel disease
RSSI: Recent small subcortical infarct
CRP: C-reactive protein
